# Modulatory Effect of Rosmarinic Acid on H_2_O_2_-Induced Adaptive Glycolytic Response in Dermal Fibroblasts

**DOI:** 10.3390/molecules28145599

**Published:** 2023-07-24

**Authors:** Suphachai Charoensin, Suwatsak Dansakda

**Affiliations:** Division of Nutrition, School of Medical Sciences, University of Phayao, Phayao 56000, Thailand; suwatsak.da@gmail.com

**Keywords:** rosmarinic acid, ROS, antioxidant, glycolysis, energy, fibroblast

## Abstract

Oxidative stress induces the adaptive response and alteration of energy metabolism across human cell types. Dermal fibroblasts shift their energy system to overload anaerobic glycolysis when exposed to sub-lethal hydrogen peroxide (H_2_O_2_). However, oxidative stress levels in the cells can be depleted by antioxidants, and such cellular changes can therefore be modulated. The present study aimed to investigate the modulatory effect of rosmarinic acid (a polyphenol antioxidant) against H_2_O_2_-induced reactive oxygen species (ROS) and the glycolytic adaptive response in fibroblasts. The results showed that H_2_O_2_ caused a significant ROS increase in the cells, and pre-treatment with rosmarinic acid (5–50 µM) decreased ROS significantly in the presence of glutathione. Rosmarinic acid modulated the adaptive response in H_2_O_2_-treated cells by decreasing glucose consumption and lactate production. The rosmarinic acid also recovered intracellular ATP and decreased NADPH production via the pentose phosphate pathway. Several glycolytic enzymes, including hexokinase-2 (HK-2), phosphofructokinase-2 (PFK-2), and lactate dehydrogenase A (LDHA), were downregulated in cells treated with rosmarinic acid. Furthermore, the key antioxidant enzymes: glutathione-disulfide reductase (GSR), glutathione peroxidase-1 (GPx-1), and peroxiredoxin-1 (Prx-1) and redox protein thioredoxin-1 (Trx-1) were upregulated in treated cells compared to control cells. To sum up, the rosmarinic acid could be used as an antioxidant against H_2_O_2_-induced adaptive responses in fibroblasts by modulating glucose metabolism, glycolytic genes, and GSH production. The present work indicates that rosmarinic acid holds promise in cell-based research applications for combating ROS and enhancing dermal fibroblast health.

## 1. Introduction

Skin aging is driven by many biochemical stimuli, including both intrinsic and extrinsic factors. The intrinsic factors involve genetics, hormones, and cellular health [1,2,3]. If there is a decreased density of dermal fibroblasts, the production of an extracellular matrix (ECM) component then tends to decline when the people age during their lifetime. This is a result of the biological aging (also known as physiological or functional aging) that occurs alongside of chronological aging in everyday life [1]. Extrinsic (environmental) factors are considered to be substantially associated with biological aging [1]. The potential insults consist of an unhealthy diet (high fat, high sugar), air pollution from particulate matter (PM), and ultraviolet (UV) radiation [2,3]. The reactive oxygen species (ROS) formed during exposure to environmental factors has been linked to imbalances in biochemical homeostasis and cellular functions [2]. The accumulated ROS production arising from physiological functions, such as the intracellular redox reaction, inflammation and immune response, mental and physical stress, and stress-response hormone cortisol release, or from environmental factors such as air pollution and UVB radiation, has the potential to disrupt the cellular processes needed to maintain biochemical activities and normal functions. Glucose metabolism is a key biochemical activity in which cells regulate the supply of sufficient chemical energy. However, this biochemical event can be deregulated systematically by accumulated ROS or oxidative stress, resulting in energy insufficiency and subsequent cellular damage and aging [4,5,6].

The exposure of environmental insults results in the generation of accumulated ROS and oxidative stress in dermal fibroblasts, which disrupts the antioxidant defense system [4]. Apart from the disruption of the cellular antioxidant system, cellular stress can interfere with mitochondrial oxidation [4,5,6]. Cellular adenosine triphosphate (ATP) is produced from the conversion of glucose to lactate in the glycolytic pathway (anaerobic glycolysis) and from oxygen consumption in mitochondrial oxidative phosphorylation (OXPHOS) [7]. The accumulated ROS further increases the adaptive response that drives glucose metabolism and the pentose phosphate pathway to produce nicotinamide adenine dinucleotide phosphate (NADPH), leading to overconsumption of it [7]. After a period of time, the cells shift energy production to anaerobic glycolysis, and this adaptive response then raises the concentration of lactic acid in the cells [5,7]. The chronic alteration of energy metabolism can contribute to fibroblast dysfunctions, such as the production of collagen and elastin, senescence, and skin aging. However, these cellular changes can be prevented or reversed by antioxidants [8,9,10,11].

**Figure 1:**
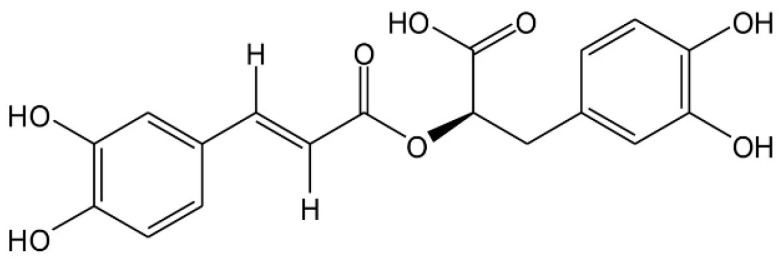
Structure of rosmarinic acid (molecular formula: C_18_H_16_O_8_), a phenolic acid derived from caffeic acid and 3,4-dihydroxyphenyl lactic acid with esterification. It is formally known as (R)-α-[[3-(3,4-dihydroxyphenyl)-1-oxo-2E-propenyl]oxy]-3,4-dihydroxy-enzenepropanoic acid.

Rosmarinic acid is an ester of caffeic acid and 3-(3,4-dihydroxyphenyl) lactic acid (Figure 1) found in herbal species including rosemary (*Rosmarinus officinalis*), basil (*Ocimum basilicum*), sage (*Salvia officinalis*), and perilla (*Perilla frutescens*) [12,13,14,15]. Rosmarinic acid is a polyphenolic compound capable of antioxidation in vitro and in vivo [13,16,17,18]. It could mitigate oxidative stress and inflammation in particulate matter (PM)-treated A549 human lung cancer cells [16]. Recent research has shown that rosmarinic acid-rich leaf extract of perilla reduced rat gastric ulcers induced by indomethacin via an anti-inflammation pathway [19]. In skin cell research, it was reported that rosmarinic acid pre-treatment protected dermal fibroblasts against an H_2_O_2_-induced inflammatory response and senescence [20]. It could stimulate collagen type I synthesis in osteogenesis imperfecta type I skin fibroblasts [21]. Moreover, it protected against the unfavorable effects of methylparaben and provided effective protection against benzophenone-3-induced alterations in skin fibroblasts [22]. However, the protective effects of rosmarinic acid on H_2_O_2_-induced ROS and adaptive responses in dermal fibroblasts have not been reported on. The present study investigated changes in glucose consumption, lactic acid, and adenosine triphosphate (ATP) upon H_2_O_2_ exposure, and the preventive effect of rosmarinic acid. Furthermore, the key glycolytic enzymes and particular antioxidant genes responding to H_2_O_2_ were also investigated. GPx-1 catalyzes the conversion of H_2_O_2_ to H_2_O using two GSH molecules. GSSG produced from GPx-1 catalysis is then reduced by GSR with NADPH as a cofactor [23]. Furthermore, Prx-1 and Trx-1 work together in the thioredoxin (Trx) system for cellular defense against oxidative stress [24]. This system provides electrons to Prx-1 to form H_2_O from H_2_O_2_ [24].

## 2. Results

### 2.1. Fibroblast Viability in Different Treatments

**Figure 2:**
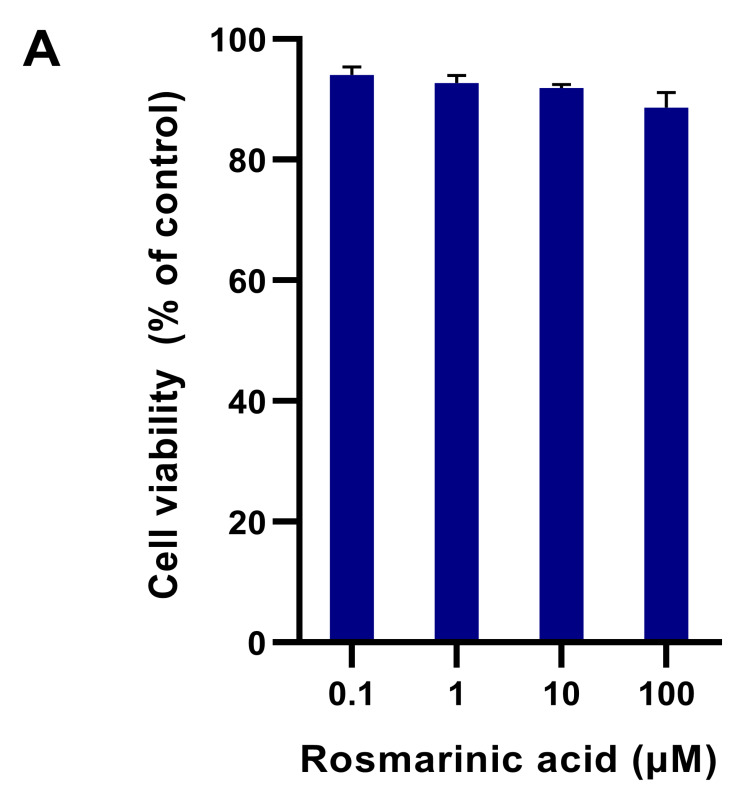

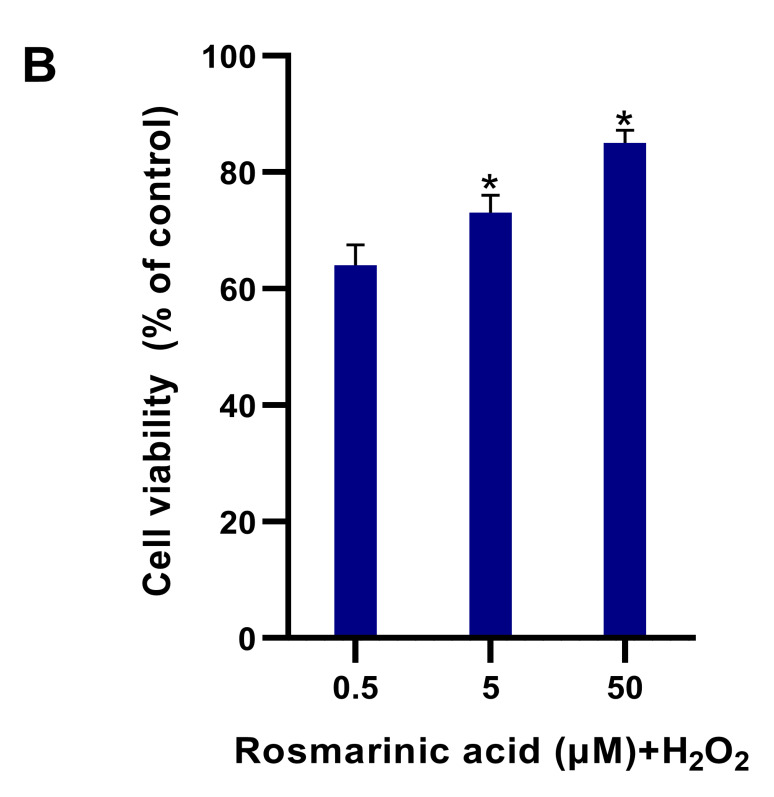
Dermal fibroblast viability. (**A**) The fibroblasts were treated with rosmarinic acid (0–100 µM). (**B**) The cells were pre-treated with rosmarinic acid (0–50 µM) for 24 hr and then treated with H_2_O_2_ (300 µM) for another 24 hr. The percentage of viable cells was shown as a mean ± SEM, * indicates *p* < 0.05 of one-way analysis of variance (ANOVA).

The dermal fibroblasts were treated with rosmarinic acid (0–100 μM). When exposed to the tested compounds, 90–98% of the treated cells survived compared to the control cells (Figure 2A). The rosmarinic acid was considered safe for further experiments. In the preventive study, the cells pre-treated with 0–50 μM rosmarinic acid survived the toxicity of H_2_O_2_ because they continued to grow up to 87% after H_2_O_2_ treatment (*p* < 0.05).

### 2.2. Effect of Rosmarinic Acid on H_2_O_2_-Induced ROS Formation in Fibroblasts

**Figure 3:**
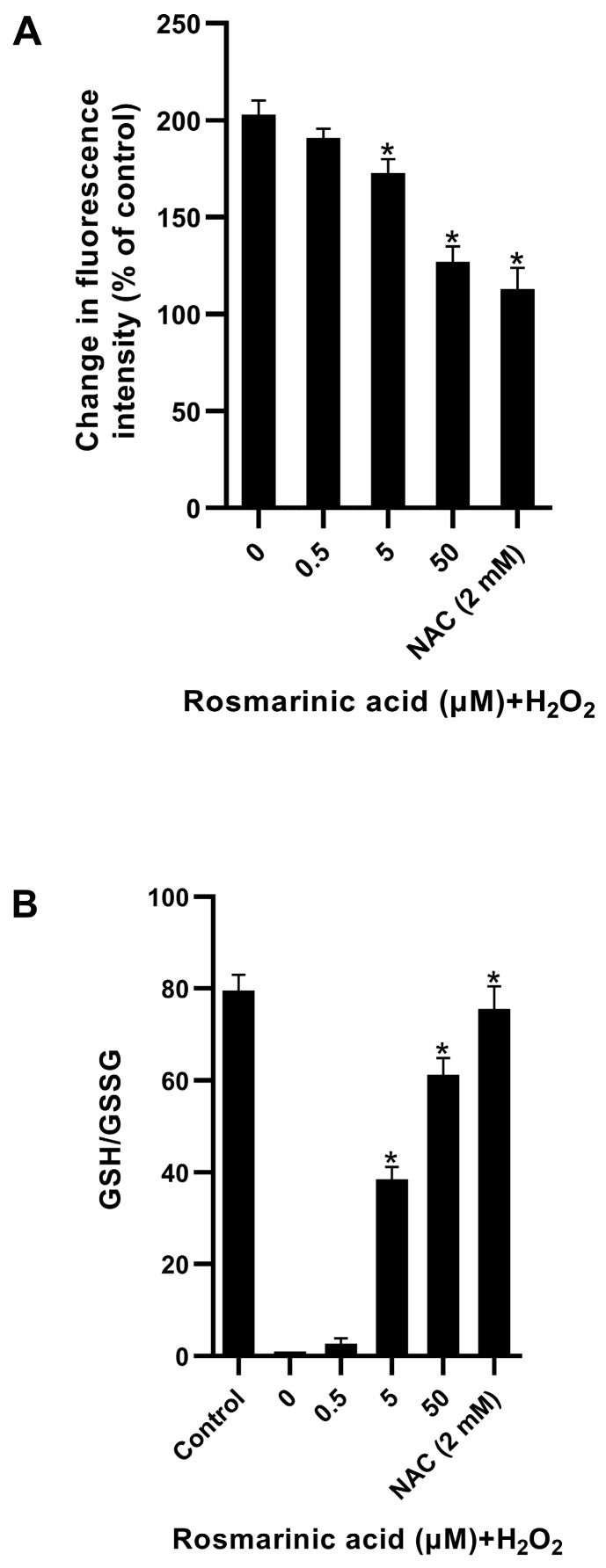
Preventive effect of rosmarinic acid against H_2_O_2_-induced ROS formation in fibroblasts: (**A**) the rosmarinic acid (0–50 μM) was pre-treated 24 hr prior to H_2_O_2_ addition. The 2, 7-dichlorofluorescein diacetate (DCFH-DA) fluorescence signals of treated cells were measured and normalized to control cells; and (**B**) the GSH/GSSG ratio from the respective rosmarinic acid pre-treatment. *N*-acetyl cysteine (NAC). Mean ± SEM from triplicate independent experiments, * indicates *p* < 0.05 of one-way analysis of variance (ANOVA).

The rosmarinic acid pre-treatment reduced the amount of ROS in the fibroblasts upon the addition of H_2_O_2_. The rosmarinic acid preventive effect was found to be significant, at 5–50 µM (Figure 3A). In this experiment, *N*-acetyl cysteine (an antioxidant) was used as a positive control. At the highest dose, the ROS level dropped as low as the *N*-acetyl cysteine pre-treatment. Both rosmarinic acid and *N*-acetyl cysteine raised the GSH/GSSG ratio (Figure 3B), which protected the cells from injury (cell viability > 90%). The results suggested that the rosmarinic acid could prevent ROS accumulation in the cells.

### 2.3. Modulatory Effect of Rosmarinic Acid on Glycolytic Adaptive Response Induced by H_2_O_2_

**Figure 4:**
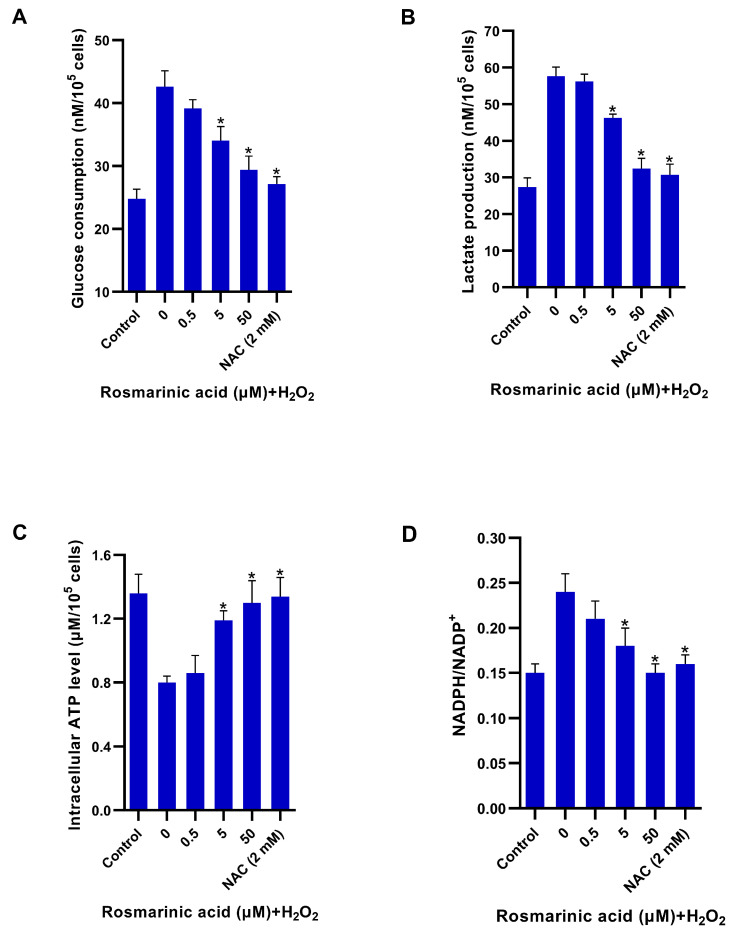
Glycolytic response in fibroblasts upon rosmarinic acid pre-treatment and H_2_O_2_ exposure. The samples from rosmarinic acid (0–50 μM) pre-treated fibroblasts were collected after H_2_O_2_ (300 μM) exposure for glucose consumption and lactate production (**A**,**B**); and intracellular adenosine triphosphate (ATP) (**C**), and ratio of NADPH/NADP^+^ in treated and control cells (**D**). *N*-acetyl cysteine (NAC). Mean ± SEM from triplicate independent experiments, * indicates *p* < 0.05 of one-way analysis of variance (ANOVA).

The effect of the rosmarinic acid pre-treatment on the H_2_O_2_-induced adaptive response was investigated. The rosmarinic acid attenuated glucose consumption significantly. The highest dose showed a comparable effect to that observed in cells treated with *N*-acetyl cysteine (Figure 4A). The reduced glucose utilization coincided with the decreased amount of lactate (Figure 4B). The rosmarinic acid recovered intracellular adenosine triphosphate (ATP) concentration, and the result was similar to that of the *N*-acetyl cysteine treatment. In addition, it reduced the ratio of NADPH/NADP^+^ (Figure 4D).

### 2.4. Effect of Rosmarinic Acid on Glycolytic Enzyme and Antioxidant Genes Expression

**Figure 5:**
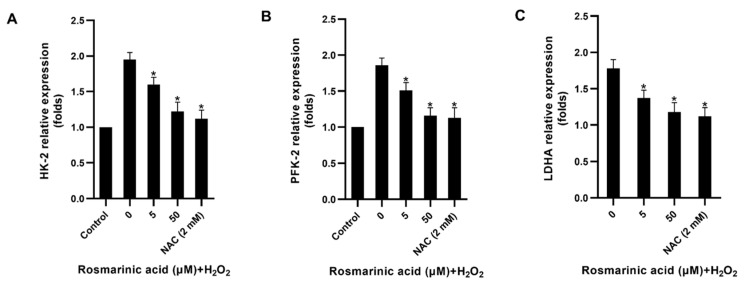
Glycolytic gene downregulation mediated by rosmarinic acid. Genes from the control and treated fibroblasts including hexokinase-2 (HK-2) (**A**); phosphofructokinase-2 (PFK-2) (**B**); and lactate dehydrogenase A (LDHA) (**C**) were quantified by quantitative real-time polymerase chain reaction. The average relative expression of the respective genes between the control and treated cells was analyzed with one-way analysis of variance (ANOVA, * as *p* < 0.05).

**Figure 6:**
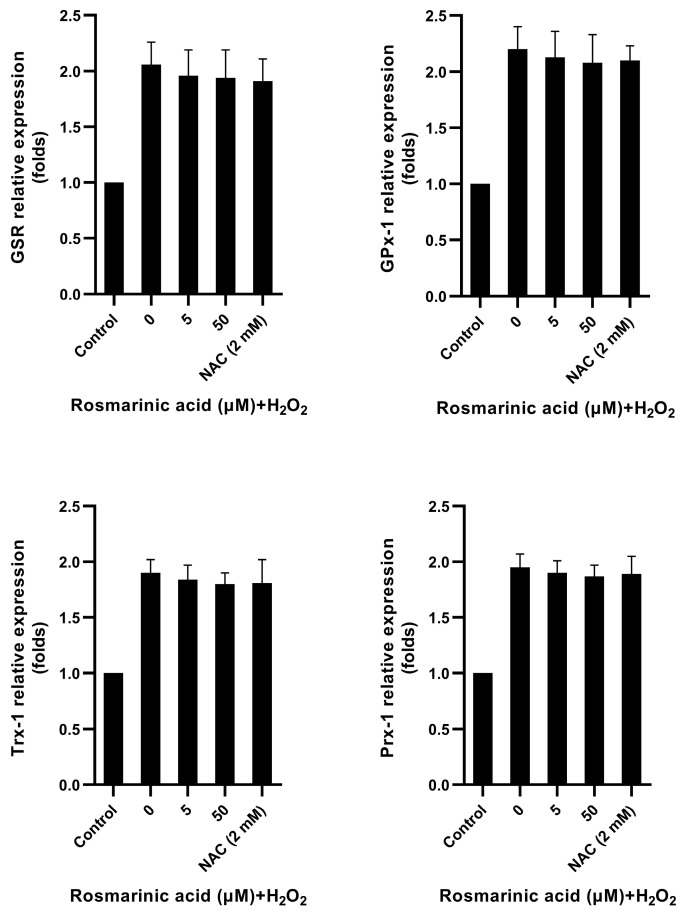
Antioxidant gene expression in response to rosmarinic acid and H_2_O_2_ treatment. The quantitative real-time polymerase chain reaction was used to measure gene expression levels of glutathione-disulfide reductase (GSR), glutathione peroxidase-1 (GPx-1), thioredoxin-1 (Trx-1), and peroxiredoxin-1 (Prx-1). Average relative expression of the genes was analyzed with one-way analysis of variance (ANOVA).

The results showed that both rosmarinic acid and *N*-acetyl cysteine significantly reduced key glycolytic enzyme genes, including hexokinase-2 (HK-2), phosphofructokinase-2 (PFK-2), and lactate dehydrogenase A (LDHA) in the H_2_O_2_-treated cells (Figure 5). Likewise, both compounds also decreased glucose consumption and lactate production (Figure 4). Lactate dehydrogenase A had no significant effect on the H_2_O_2_-treated cells, and all the antioxidant genes were upregulated upon treatment (Figure 6).

## 3. Discussion

During normal mitochondria oxidation of most cell types, OXPHOS accounts for more than 80% of cellular ATP production [25]. The ATP rate index demonstrates that fibroblasts switch to more aerobic metabolism when fibroblasts have a lower ATP rate index than other cell lines. This was confirmed in recent work by Algieri and colleagues on the role of mitochondrial OXPHOS, which is the leading energy supplier for normal fibroblasts, as well as preadipocytes [26]. They pointed out that glycolysis was inhibited in type-2 familial partial lipodystrophy (FPLD2, a lamin A/C mutation) fibroblast cell lines, and that this pathological condition was related to impaired cell metabolism. The amount of ATP produced in the fibroblasts was higher than in normal fibroblasts and preadipocytes with respect to FPLD2 [26]. The cellular oxidative stress model was established according to the investigation of sub-lethal H_2_O_2_ exposure to normal human dermal fibroblasts and the primary culture of dermal fibroblasts from myoclonic epilepsy with ragged red fibers or MERRF syndrome (one of the mitochondrial diseases associated with an A to G transition at nucleotide position 8344 (A8344G mutation) in the tRNA^Lys^ gene of mtDNA) [7]. Molavian and colleagues demonstrated that the disruption of glycolysis by H_2_O_2_ resulted in a drop in ATP [5]. The energy metabolism shift induced by oxidative stress (an imbalance between oxidant and antioxidant) was thoroughly investigated to understand how to prevent or attenuate cellular dysfunctions [5,7,8]. The cellular adaptation to such stress requires the regulation of glycolysis and the pentose phosphate pathway to support NADPH-dependent antioxidant defense [7,8,27]. The dermal fibroblasts were more dependent on anaerobic glycolysis when exposed to sub-lethal H_2_O_2_ [4,28]. The excess amount of reactive oxygen species (ROS) from intracellular redox reactions in response to H_2_O_2_ affects glycolytic enzymes and promotes flux into the pentose phosphate pathway [7,27]. The modulation of redox homeostasis by reducing ROS accumulation and inducing an antioxidant enzyme system is therefore a strategy to tackle ROS-mediated metabolic shifts and aging cells [1,3,7,28].

In this study, rosmarinic acid (a plant-derived polyphenolic antioxidant) demonstrated a protective effect against ROS in fibroblasts (Figure 3). The pre-treatment of rosmarinic acid could raise intracellular GSH to protect cells from ROS. It was reported that rosmarinic acid possesses in vitro and in vivo antioxidant activities and induces antioxidant enzymes through the Nrf2 pathway [23,29,30]. H_2_O_2_ can be harmful if its intracellular concentration is rather high, and this can disrupt glucose consumption and trigger non-oxidative glycolysis (pyruvate → lactate) [5,7,27]. The rosmarinic acid pre-treatment could improve glucose utilization and reduce lactate formation in a comparable manner to *N*-acetyl cysteine (Figure 4A,B). In response to ROS, NADPH is produced more via the pentose phosphate pathway [5,7,27]. Both rosmarinic acid and *N*-acetyl cysteine, however, modulated the flux and reduced NADPH content (Figure 4D). Nevertheless, the cellular NADPH might be oxidized during GSH restoration. In line with previous reports, the *N*-acetyl cysteine has a scavenging activity against ROS through thiol-containing cysteine, and it is also used as a substrate for endogenous GSH production [10]. The results indicated that rosmarinic acid is able to shape cellular glucose utilization, glycolysis, and GSH.

Key glycolytic genes in fibroblasts were also investigated. H_2_O_2_ increased glucose utilization, and the respective genes’ expression [5,7,8]. The pre-treatment of rosmarinic acid prior to H_2_O_2_ exposure decreased the expression of HK-2, PFK-2, and LDHA (Figure 5), and the *N*-acetyl cysteine showed a similar effect. In line with the previous work, it was reported that H_2_O_2_ can cause oxidative stress and mediate the increase of glycolytic flux in dermal fibroblasts by upregulating the key glycolytic enzyme genes [7]. Such an effect could be reversed by *N*-acetyl cysteine, resulting in decreased glucose and lactate contents in the cells [7]. Due to a strong antioxidant capacity, rosmarinic acid reduced ROS accumulation and it could activate various antioxidant genes through the Nrf2 pathway [20,23,29,31]. However, two glutathione-recycling enzymes, GSR and GPx-1, Trx-1, and Prx-1 in H_2_O_2_-treated cells were not significantly affected by pre-treatment with rosmarinic acid. Thus, the results indicate that rosmarinic acid could mitigate ROS due to its polyphenol-related antioxidant activity. Based on the present findings, the results were limited to the gene expression level. The protein expression of the observed antioxidant enzymes and glycolytic pathway might be interesting points of study for further investigations. In addition, the fibroblast mitochondria should be considered as a target as well. 

## 4. Materials and Methods

### 4.1. Chemicals

Analytical grade chemicals were used in this work. H_2_O_2_ was purchased from Merck Co. (Darmstadt, Germany). The SuperKine™ cell counting kit-8 (CCK-8, water-soluble tetrazolium salt-(WST-8)) and protein quantification (Bradford reagent) kit were from Abbkine, Inc., Wuhan, China. Rosmarinic acid and *N*-acetyl cysteine (NAC) were purchased from Sigma Chemical Co. (St. Louis, MO, USA). Eagle’s minimum essential medium (EMEM), fetal bovine serum (FBS), and antibiotic-antimycotic reagent for cell culture were purchased from Thermo Fisher Scientific Inc. (Waltham, MA, USA). Primers were from Macrogen, Inc. (Seoul, Republic of Korea). TRIzol™ reagent (Ambion^®^) was from Thermo Fisher Scientific Inc. (Waltham, MA, USA). The SensiFAST™ SYBR^®^ One-step kit was purchased from Meridian Bioscience Inc. (Cincinnati, OH, USA). The amount of ROS, adenosine triphosphate (ATP), glucose, lactate, nicotinamide adenine dinucleotide phosphate (NADP^+^ and NADPH), and glutathione (GSSG and GSH) were measured using biochemical assay kits from Dojindo Laboratories (Kumamoto, Japan). 

### 4.2. Cell Culture and Treatments

Human dermal fibroblasts (BJ, CRL-2522™) were purchased from The American Type Culture Collection (ATCC^®^) (Manassas, VA, USA). BJ fibroblasts were previously selected for investigating molecular mechanism of the regulation of energy metabolism under oxidative stress and for studying senescence-associated glycolytic overload and identifying antisenescence activity of plant-derived natural compounds [7,8]. Thus, it was considered the proper cell model for the present study. The fibroblasts were cultured in Eagle’s minimum essential medium containing 10% FBS, 1% non-essential amino acids, 2 mM L-glutamine, 1 mM sodium pyruvate, and 1500 mg/L of sodium bicarbonate. The cells were maintained at 37 °C, 5% CO_2_ and 95% air and were used for the assays when the population reached 80% confluency [9]. The cells were inoculated in 1 × 10^3^ cells/well for a 96-well plate for the cytotoxicity test of rosmarinic acid. For other assays, 1 × 10^5^ cells were seeded into vessels [7,9]. Either rosmarinic acid or *N*-acetyl cysteine (NAC, a standard antioxidant) were pre-treated 24 hr prior to H_2_O_2_ treatment to investigate the prevention of both compounds on H_2_O_2_-induced ROS accumulation, energy adaptive response, and the respective gene changes. *N*-acetyl cysteine (2 mM) was used as a positive control. 

### 4.3. Cytotoxicity Test 

The fibroblasts treated with either rosmarinic acid (0–100 µM) or rosmarinic acid (0–50 µM) + H_2_O_2_ (300 µM) were tested for cell proliferation after being incubated for 48 hr at 37 °C, 5% CO_2_ and 95% air. The 10 µL of WST-8 (SuperKine^™^ cell counting kit-8, Abbkine, Inc., Wuhan, China) was added to each well, and the plates were placed back in the CO_2_ incubator. The WST-8 tetrazolium salt was reduced by cellular mitochondrial dehydrogenases to an orange formazan product that was soluble in the culture medium in the presence of electron-coupled reagents. Within 4 hr of incubation with the WST-8, the amount of formazan produced was directly proportional to the number of proliferating fibroblasts measured at 450 nm (Biotek Cytation 5 multi-mode reader, Santa Clara, CA, USA). The results were calculated and expressed as average percentages of the control cells.

### 4.4. Reactive Oxygen Species (ROS) Measurement

ROS play an essential role in cell-signaling pathways, whereas an increased amount of ROS is associated with oxidative stress and cell aging. After pre-treatment, the cultured media from untreated and treated cells were replaced with 2, 7-dichlorodihydrofluorescein diacetate or DCFH-DA (Dojindo Laboratories, Kumamoto, Japan) and left for 30 min. The cells were washed twice with Hanks’ balanced salt solution (HBSS) and then cultured with H_2_O_2_. The fluorescence signals were measured at λ_ex_ = 505 nm, λ_em_ = 525 nm (Biotek Cytation 5 multi-mode reader, Santa Clara, CA, USA).

### 4.5. Adenosine Triphosphate (ATP) Measurement

Following the Dojindo’s ATP assay kit, ATP levels were measured with a luminescent reaction. In principle, the healthy, living fibroblasts synthesize ATP by both glycolysis and mitochondrial oxidative phosphorylation. The latter generates most cellular ATP (80% on average), and the dysfunction reduces ATP levels in the cells. When the amount of ROS in the cells exceeds in response to intrinsic and extrinsic factors, the cell’s ability to combat it starts to reduce. To study the prevention of such a phenomenon, the cells were pre-treated with rosmarinic acid (0–50 µM) or *N*-acetyl cysteine (2 mM) for 24 hr followed by H_2_O_2_ for another 24 hr. The culture medium was replaced with the ATP working solution (90 µL) and incubated at 25 °C for 10 min in a microplate reader. Then, the luminescent signals were recorded (Biotek Cytation 5 multi-mode reader, Santa Clara, CA, USA). The intracellular ATP was calculated using a calibration curve (0–2.5 µM). 

### 4.6. Determination of Glucose, Lactate, and NADPH/NADP^+^

Metabolic activity changes upon H_2_O_2_ and rosmarinic acid and *N*-acetyl cysteine treatment were measured using a glucose assay kit (Dojindo Laboratories, Kumamoto, Japan). The cell-culture supernatants were collected after incubation was completed and diluted with deionized water. The standard glucose concentrations for the calibration curve ranged from 0–0.5 mM. The amounts of supernatants from untreated and treated cells or standard glucose (50 µL) were mixed with a working solution (50 µL) containing enzymes and water-soluble formazan. The colorimetric reaction was measured at 450 nm (Biotek Cytation 5 multi-mode reader, Santa Clara, CA, USA). The amounts of lactate in the respective supernatants were determined with a lactate assay kit-WST (Dojindo Laboratories, Kumamoto, Japan). The absorbance values of sample supernatants measured at 450 nm were compared to the standard curve of lactate (0–1.0 mM). NADP^+^ and NADPH in the cell lysates were quantified with the lactate assay kit-WST (Dojindo Laboratories, Kumamoto, Japan). The absorbance values were measured at 450 nm nm (Biotek Cytation 5 multi-mode reader, Santa Clara, CA, USA). The amount of NADP^+^ was calculated by subtracting the amount of NADPH from the amount of total NADPH/NADP^+^ and normalized with the cell numbers. The results were represented as an average ratio.

A cell-count normalization kit (Dojindo Laboratories, Kumamoto, Japan) was used to correct the measured values and to obtain the exact quantitative values. The kit employs a DNA-binding probe (Hoechst 33342). After binding to the DNA groove, the probe emits bright blue fluorescence at 461 nm upon excitation at 350 nm. After removing the cell-culture supernatants, the sample cells from control and treated cells were washed with phosphate buffer saline, pH 7.4, then added to the working solution (250 µL/well) consisting of a staining solution and quenching buffer. After 30-min incubation in 5% CO_2_ incubator at 37 °C, the solution in each well was replaced with a dilution buffer (250 µL). The fluorescence intensity was read using the Biotek Cytation 5 multi-mode reader (λ_ex_ = 350 nm, λ_em_ = 461 nm) (Santa Clara, CA, USA). The cell number and fluorescence signal intensity were plotted to create a calibration curve (0–4.0 × 10^5^). The number of control and treated cells was calculated.

### 4.7. GSH/GSSG Quantification

The control and treated cell lysates were prepared by mixing cells with 10 mM HCl (80 µL) and 5% sulfosalicylic acid (SSA, 20 µL). After 10 min of centrifugation (8000× *g*), the supernatants were collected. For GSSG measurement, the masking solution (4 µL) was added to a sample (200 µL) followed by a DTNB-containing buffer solution (60 µL) and incubated at 37 °C for 60 min. The unmasked samples were also prepared for total glutathione determination. Then, two more solutions were added to the mixtures, a substrate working solution (60 µL) and an enzyme/coenzyme working solution (60 µL). After being incubated at 37 °C for 10 min, the absorbance of all the reaction mixtures was read at 405 nm with a Biotek Cytation 5 multi-mode reader (Santa Clara, CA, USA). The GSSG content was calculated using a GSSG calibration curve (0–25.0 µM). The amount of total glutathione (GSSG+GSH) was determined using a GSH curve (0–50.0 µM). The GSH concentration was calculated using the equation, GSH = total glutathione − GSSG × 2). 

### 4.8. Ribonucleic Acid (RNA) Isolation and Quantitative Real-Time Polymerase Chain Reaction 

The RNA of the untreated and treated cells was isolated using TRIzol™ reagent (Ambion^®^, Thermo Fisher Scientific Inc., Waltham, MA, USA). After chloroform extraction, the RNA suspended in the aqueous layer was precipitated with isopropanol. Isolated RNA was re-suspended in ultrapure water and kept in a −70 °C freezer. The expression of genes (Table 1) was quantified using the SensiFAST™ SYBR^®^ One-step kit (Meridian Bioscience Inc., Cincinnati, OH, USA). Following the manufacturer’s protocol, an RT-qPCR reaction mix (20 µL) consisting of SensiFAST™ SYBR^®^ one-step mix (10 µL), reverse transcriptase (0.2 µL), RiboSafe RNase Inhibitor (0.4 µL), DEPC-H_2_O (3.8 µL), and template (4 µL) was run in QIAquant 96 5plex (Qiagen, Düsseldorf, Germany). For comparison of expression levels, the expression of each gene was normalized to *β*-actin and shown as a relative expression (2^−ΔΔCt^, ΔΔCt = ΔCt_target_ − ΔCt*_β_*_-actin_).

### 4.9. Protein Quantification

Based on the Bradford assay, the protein quantification (Abbkine, Inc., Wuhan, China) kit utilizes an improved Coomassie blue G-250, which forms a blue complex in the presence of protein. The intensity of the blue complex is proportional to the amount of protein in the sample. Briefly, the culture media were removed from the control and treated cells, then the cells were lysed with a lysis buffer containing 10 mM Tris, 100 mM NaCl, 1 mM EDTA, 1 mM EGTA, 1 mM NaF, 20 mM Na_4_P_2_O_7_, 2 mM Na_3_VO_4_, 1% Triton X-100, 10% glycerol, 0.1% SDS, and 0.5% deoxycholate. After completing lysis, the cell lysates were centrifuged at 8000× *g* for 30 min 4 °C, and the supernatants were collected for protein assay. The diluted sample supernatants were mixed with Bradford working solution (200 µL) in a 96-well plate and left for 5 min. When the Coomassie dye-bound protein was placed in an acidic medium, an immediate shift in absorption maximum occurred from 465 nm to 595 nm with a concomitant color change from brown to blue. The optical density was measured at 595 nm (Biotek Cytation 5 multi-mode reader, Santa Clara, CA, USA). The amount of protein in each sample supernatant was calculated with a bovine serum albumin (BSA) standard curve (0–1000 µg/mL).

### 4.10. Statistical Analysis

The experimental data obtained from triplicate independent experiments were expressed as the mean ± standard error of means (SEM). The difference between untreated and treated groups was analyzed by one-way analysis of variance (ANOVA). A *p*-value less than 0.05 was considered statistically significant.

## 5. Conclusions

The present work demonstrated that rosmarinic acid regulated the glycolytic response and reduced oxidative stress in H_2_O_2_-treated fibroblasts. Based on its protective activity, it is applicable in the research and development of cell-based supplements for the prevention of stress-mediated cellular energy disruptions.

## Figures and Tables

**Table 1:** Primer sequences for qPCR.

Target Genes	Forward Primers	Reverse Primers
HK-2	GAGTTTGACCTGGATGTGGTTGC	CCTCCATGTAGCAGGCATTGCT
PFK-2	TACCGACCTCTTGACCCAGACA	TAAATGGTGCGAGGCTGGACGT
LDHA	GGATCTCCAACATGGCAGCCTT	AGACGGCTTTCTCCCTCTTGCT
GSR	TATGTGAGCCGCCTGAATGCCA	CACTGACCTCTATTGTGGGCTTG
GPx-1	GTGCTCGGCTTCCCGTGCAAC	CTCGAAGAGCATGAAGTTGGGC
Trx-1	GTAGTTGACTTCTCAGCCACGTG	CTGACAGTCATCCACATCTACTTC
Prx-1	CTGCCAAGTGATTGGTGCTTCTG	AATGGTGCGCTTCGGGTCTGAT
*β*-actin	CACCATTGGCAATGAGCGGTTC	AGGTCTTTGCGGATGTCCACGT

## Data Availability

Not applicable.

## Sample Availability

Samples of the compounds are not available from the authors.
